# Unanticipated Geochemical and Microbial Community Structure under Seasonal Ice Cover in a Dilute, Dimictic Arctic Lake

**DOI:** 10.3389/fmicb.2016.01035

**Published:** 2016-07-05

**Authors:** Ursel M. E. Schütte, Sarah B. Cadieux, Chris Hemmerich, Lisa M. Pratt, Jeffrey R. White

**Affiliations:** ^1^Integrated Program in the Environment, Indiana University, BloomingtonIN, USA; ^2^Institute of Arctic Biology, University of Alaska Fairbanks, FairbanksAK, USA; ^3^Department of Geological Sciences, Indiana University, BloomingtonIN, USA; ^4^University of Illinois at Chicago, ChicagoIL, USA; ^5^Center for Genomics and Bioinformatics, Indiana University, BloomingtonIN, USA; ^6^School of Public and Environmental Affairs, Indiana University, BloomingtonIN, USA

**Keywords:** microbial communities, geochemistry, Arctic, lakes, seasonally ice-covered

## Abstract

Despite most lakes in the Arctic being perennially or seasonally frozen for at least 40% of the year, little is known about microbial communities and nutrient cycling under ice cover. We assessed the vertical microbial community distribution and geochemical composition in early spring under ice in a seasonally ice-covered lake in southwest Greenland using amplicon-based sequencing that targeted 16S rRNA genes and using a combination of field and laboratory aqueous geochemical methods. Microbial communities changed consistently with changes in geochemistry. Composition of the abundant members responded strongly to redox conditions, shifting downward from a predominantly heterotrophic aerobic community in the suboxic waters to a heterotrophic anaerobic community in the anoxic waters. Operational taxonomic units (OTUs) of Sporichthyaceae, Comamonadaceae, and the SAR11 Clade had higher relative abundances above the oxycline and OTUs within the genus *Methylobacter*, the phylum Lentisphaerae, and purple sulfur bacteria (PSB) below the oxycline. Notably, a 13-fold increase in sulfide at the oxycline was reflected in an increase and change in community composition of potential sulfur oxidizers. Purple non-sulfur bacteria were present above the oxycline and green sulfur bacteria and PSB coexisted below the oxycline, however, PSB were most abundant. For the first time we show the importance of PSB as potential sulfur oxidizers in an Arctic dimictic lake.

## Introduction

Lakes are important participants in the global carbon cycle (Tranvik et al., 2009), processing carbon from terrestrial and aquatic ecosystems and contributing 6–16% of the total natural methane (CH_4_) emissions (Bastviken et al., 2008; Borrel et al., 2011). Despite the fact that most lakes in the Arctic are perennially or seasonally frozen (Walsh et al., 1998; Comeau et al., 2012) very little information is available on microbial communities and nutrient cycling under ice cover (Bertilsson et al., 2013). The ice-covered season affects the ecology and metabolic characteristics of microbial communities and their contributions to the food web and biogeochemical cycling throughout the year. A reduction in ice coverage and related transition of perennially frozen lakes to seasonally frozen lakes due to warming temperatures underscores the importance of understanding microbiomes under ice for improved prediction of the responses of Arctic lakes to rapidly warming climate.

Few studies have documented prokaryotic communities under ice cover. Microbial biomass is lower under ice cover compared to the ice-free period (Personnic et al., 2009; Twiss et al., 2012) due to lower temperatures, reduced nutrient inputs and decreases in the quality of organic substrates because of lower autochthonous production and limited inputs of labile terrestrial organic matter (Tulonen, 1993; Tulonen et al., 1994; Bergström and Jansson, 2000). However, microbial communities can actively grow during winter (Bertilsson et al., 2013). Beall et al. (2015) found diverse microbial communities in Lake Erie under ice cover including among others Verrucomicrobia, Proteobacteria, and Bacteroidetes. Similarly Glatz et al. (2006) described a diverse community in permanently frozen lakes in Antarctica comprising Proteobacteria, Actinobacteria, Bacteroidetes, and Planctomycetes. These diverse microbial communities have been shown to change systematically during the ice-covered season.

Studies on linking microbial communities with geochemistry in ice-covered lakes are limited, with most focusing on perennially frozen lakes. In two Siberian seasonally ice-covered lakes, Rogozin et al. (2009) found high abundances of purple sulfur bacteria (PBS) at ice on and greatly varying abundances throughout the ice-covered season due to variations in light availability and redox conditions. In meromictic perennially frozen lakes green sulfur bacteria (GSB) are commonly found and PSB are not detected (Lauro et al., 2011). Both PSB and GSB are important sulfur oxidizers in these systems that utilize light. The relative abundance between PSB and GSB can change by season based on changes in physicochemical gradients and light availability (Abella et al., 1979; Blankenship et al., 1995). PSB and GSB differ in their pigment content; PSB use bacteriochlorophyll *a* (BChl *a*), while GSB use BChl *c, d*, and *e* (Guerrero et al., 1985; Stomp et al., 2007; Rogozin et al., 2009). This divergence in pigment composition of phototrophic microorganisms and the associated absorbance characteristics appears to support species coexistence and biodiversity in aquatic environments (Stomp et al., 2004, 2007). Villaescusa et al. (2010) and Bielewicz et al. (2011) attributed changes in microbial community composition in permanently frozen lakes in Antarctica to changes in overall productivity, stratification, and nutrient availability. Comeau et al. (2012) observed that bacterial community composition differed between two time points of ice coverage in a high Arctic lake in Canada, and that microbial communities were stratified by depth with good agreement to chemical gradients.

Here, we assessed the vertical microbial community distribution and geochemical composition under ice in a seasonally ice-covered lake in Southwest Greenland. During preliminary sampling under ice cover in 2013, we detected a diverse community of PSB forming a pinkish layer at a depth of 6 m and identified the most abundant operational taxonomic unit (OTU, 39%) as *Lamprocystis*. The dense community of PSB corresponded with both a sharp redox gradient and an increase in sulfide (ΣH_2_S) and methane (CH_4_) concentrations. In 2014, using amplicon-based sequencing targeting the 16S rRNA genes and aqueous geochemical approaches, we found a highly diverse microbial community throughout the water column and very good spatial agreement between CH_4_, sulfur, and nitrogen gradients and changes in the microbial community composition. In addition, we show for the first time PSB to be the most abundant potential sulfur oxidizers in a dimictic, Arctic lake compared to green sulfur bacteria (GSB) and purple non-sulfur bacteria (PNSB) that have been shown to be the more abundant sulfur oxidizers in permanently frozen lakes in the Arctic (Comeau et al., 2012) and Antarctica (Karr et al., 2003).

## Materials and Methods

### Site Description

The region of southwest Greenland between 66° and 68°N is characterized by low continental climate and is strongly influenced by the high-pressure system over the Greenland Ice Sheet (GIS; Bennike, 2000). Mean annual air temperature at the Kangerlussuaq weather station from 1974 to 2012 was -6°C. During the warm season of late May to early September, mean temperatures exceed +10°C and peak to +20°C in July. The cold season extends from November to late March, with average daily temperature below -9°C. Precipitation is minimal, with annual precipitation <150 mm yr^-1^, mostly in the summer months. Bedrock in the region is composed of complexly deformed Archean felsic gneisses (Jensen et al., 2002). The terrestrial plant community is primarily dwarf-shrub tundra, dominated by species such as *Salix arctica, Salix glauca, Betula nana, Vaccinium uliginosum, Sphagnum* spp., and various grasses and sedges.

The area has continuous permafrost <50 cm below the surface extending down to 150–500 m depth (Jørgensen and Andreasen, 2007). Lakes in the region are frozen for ∼10 months of the year, with ice-out occurring by mid-June and re-freezing in mid-September (Anderson and Brodersen, 2001). As a result of minimal precipitation, lakes are supplied with water mainly through contributions from melting of the limited snowpack. Through-going drainage is absent and groundwater seepage into the small lakes is assumed to be limited due to continuous permafrost. Consequently, the small lakes of the region are primarily isolated evaporitic basins at this point in time.

### Potentilla Lake

Potentilla lake (67° 04′ 51.64″N, 50° 21′ 17.14″W) is located ∼6 km from the terminal moraine of the Russell Glacier (Colcord et al., 2015; Webster et al., 2015; Cadieux et al., 2016; Goldman et al., 2016). It is a relatively small lake, with a surface area of 1.6 ha, volume of 56,000 m^3^, and maximum depth of 7.8 m and it is ice-covered for about 9 months of the year. The lake basin is elliptical and asymmetric. Potentilla is a dilute, oligotrophic lake, with mean conductivity of 170 μS cm^-1^ and mean dissolved organic carbon of 11 mg L^-1^ (Cadieux et al., 2016). Under open-water conditions in 2013, the pH ranged from 7.2 to 9.0. Currently, Potentilla is a hydrologically closed basin, with no direct inflow or outflow channel, although there is evidence of overflow during previous wetter periods. While many lakes in the Kangerlussuaq region are cold monomictic (Anderson et al., 2001), Potentilla lake is a dimictic lake.

### Sampling and *In Situ* Analysis

Sampling for this study took place in April 2014, at the end of winter stratification when the lake was covered with ∼2 m of ice and ∼20 cm of snow. A hole (∼30 cm in diameter) was augered through the ice above maximum depth (*Z*_max_). Profiles of temperature (T, °C), dissolved oxygen (DO, %), pH, and oxidation-redox potential (ORP, mV) were measured using a YSI 6093 Data Sonde (Yellow Springs Inc., Yellow Springs, OH, USA). A Li-Cor LI193 Spherical Quantum Sensor (Li-Cor, Lincoln, NE, USA) was used to measure photosynthetically active radiation (PAR, μmol m^-2^ s^-1^) attenuation with depth. Water samples were taken with an electronic submersible pump at 0.5 m depth intervals from just below the ice at 2.5 to 7.0 m (nine sample intervals). For chemical analysis, samples were filtered within 24 h of collection using a series of Whatman GF/F glass microfiber filters and Millipore membrane filters and frozen immediately until analysis. For microbial analysis, 1 L of water was collected into sterile HDPE Nalgene bottles and filtered through Millipore Isopore Membrane Filters (0.22 μm) that were frozen immediately and transferred to a -80°C freezer until analysis.

### Chemical Analysis

Total dissolved sulfide concentrations (ΣH_2_S) in the water column were determined gravimetrically by precipitation of H_2_S(aq) and HS^-^(aq) as cadmium sulfide (CdS) precipitate. Ten liters of water were collected in Nalgene carboys, and fixed in the field immediately upon collection by using pre-charged sample containers with 200 mL of supersaturated cadmium chloride (CdCl_2_) solution (Szynkiewicz et al., 2009). The precipitated CdS was then extracted using an acid-volatile sulfide (AVS) technique (Szynkiewicz et al., 2009) and the S precipitated as silver sulfide (Ag_2_S). Sulfide concentrations were then determined by the gravimetric yields of collected Ag_2_S, and assuming a stoichiometry of H_2_S/HS^-^:Ag_2_S of 1. Minimum detection limits using cadmium precipitation are 0.5 μM based upon laboratory experiments with pure H_2_S and water. Concentrations of sulfate (SO_4_^2-^), nitrite (NO_2_^-^), nitrate (NO_3_^-^) and ammonium (NH_4_^+^) were analyzed using a Dionex ICS 2000 Ion Chromatograph using a CS12A analytical cation column, CSRS 300 4 mm suppressor, and 20 mM methanesulfonic acid eluent for cations and AS11-HC analytical anion column, ASRS 4 mm suppressor and 30 mM potassium hydroxide eluent for anions. Minimum detection limits were NH_4_^+^= 0.5, NO_2_^-^= 0.6, SO_4_^2-^ = 1.8, and NO_3_^-^ = 0.2 μeq L^-1^.

Water samples for dissolved CH_4_ in the water column were immediately stripped in the field using a headspace-equilibrium technique (Westendorp, 1985) to extract CH_4_ from water using a 1 L Erlenmeyer flask. Headspace gas in the flask was displaced into a Cali-5-Bond bag using surficial lake water. The concentrations of dissolved aqueous CH_4_ were measured using a Los Gatos Research (LGR) Methane Carbon Isotope Analyzer (MCIA; LGR, Mountain View, CA, USA) that was operated at the field lab in Kangerlussuaq (Cadieux et al., 2016). All samples were processed within 24 h of collection. The total concentration of CH_4_ in each sample was corrected for dilution and calculated from the sum of the measured headspace partial pressure and the dissolved CH_4_ remaining after gas stripping, according to Henry’s law using values from Lide and Fredrikse (1995) and Cadieux et al. (2016). Instrumental uncertainty on CH_4_ concentrations from the MCIA was ± 0.5 ppmv, which is one standard deviation of the values for gas standards analyzed during sample runs.

### Microbial Community Analysis

Genomic DNA and RNA were co-extracted using the PowerWater RNA isolation kit (MO BIO Laboratories, Carlsbad, CA, USA). As we were interested in describing changes in community composition we only used the genomic DNA. Amplification of the 16S rRNA genes was performed using the forward primer 515f and a barcoded reverse primer 806r (Caporaso et al., 2012^1^). Each reaction was 25 μL in volume, with 5 μL 5× HF Buffer (New England Biolabs, Ipswich, MA, USA), 1.5 μL of 50 μM MgCl_2_, 0.5 μL 10 mM dNTPs, 0.5 μL of each 10 μM primer (Integrated DNA Technologies, Coralville, IA, USA) 0.25 μL of 2000 U/mL Phusion DNA Polymerase (New England Biolabs, Ipswich, MA, USA), 10–20 ng of DNA, and 15.75 μL PCR water. Amplification was performed using an initial incubation step at 94°C for 3 min followed by 35 cycles of 94°C for 45 s, 50°C for 1 min, 72°C for 1.5 min, and a final extensions step at 72°C for 10 min. PCR amplicons were cleaned using the PCR purification kit (QIAGEN, Valencia, CA, USA). Amplicons were pooled in an equimolar mixture and the pool was cleaned an additional time using the Agencourt AMPure XP (Beckman Coulter, Brea, CA). Sequences were generated on the MiSeq Illumina platform using MiSeq Reagent Kit v2 (Illumina Inc, San Diego, CA, USA) and custom-made sequencing primers ^2^. Quality control and analysis of the sequences were performed following the Mothur MiSeq SOP (Kozich et al., 2013).

### Sequence Analysis

Quality control of the sequences was performed following the Mothur MiSeq SOP (Kozich et al., 2013) with the following settings. The MiSeq sequencer sequences each PCR amplicon from both ends giving a Read 1 and Read 2. For each read pair, a contig was assembled from Read 1 and the reverse complement of Read 2. If a base only existed in one of the reads and had a quality score (Q score) less than 25, it was marked as ambiguous. If the reads disagreed on a base and the Q score disparity was six or more, the higher quality base was used. Otherwise the base was marked as ambiguous. If the contig was longer than 260 base pairs in length, or contained one or more ambiguous bases, it was discarded. The contigs were then aligned to the SILVA 16S reference alignment http://blog.mothur.org/2014/08/08/SILVA-v119-reference-files/ and reads that did not map between position 13859 and 23447 of the alignment were discarded. We then used the Mothur pre.cluster command to combine sequences within two base pairs of a more abundant sequence to further reduce read error. We then used the chimera.uchime command in Mothur to remove chimeras from each sample with the setting dereplicate = t so that if a sequence was marked as a chimera in some but not all samples, it was only removed from samples in which it was determined to be a chimera. To remove sequences other than Bacteria or Archaea, we classified each contig sequence against the RDP PDS 16S training set using the classify.seqs Mothur command and removed any sequence classified as Chloroplast, Mitochondria, unknown, or Eukaryota. We clustered the remaining sequences using average linkage into OTUs using Mothur with a similarity cutoff of 97% (Schloss et al., 2009). The OTUs obtained through Mothur were classified using both the SILVA database (Quast et al., 2013) and the RDP PDS 16S training set. The taxonomic identification was consistent between both databases, but in a few cases SILVA provided classification with finer phylogenetic resolution. Thus, the data presented here is based on the classification obtained with the SILVA database. Mothur classifies all sequences and reports the most common result for each OTU. Each level of classification is scored by the percentage of reads within the OTU that agree. We truncated all OTU taxonomic identifications to the most specific level with at least 99% consensus. We used the proportions of each OTU within each sample for downstream analysis to account for differences in number of sequences obtained across samples. Sequences were submitted to the NCBI Sequence Read Archive, accession number SRP075219.

We calculated Chao1 richness estimator and inverse Simpson diversity for all samples using the “summary.single” command in Mothur. This command rarefies all samples to the sequence count of the smallest sample. This process is repeated 1000 times and the average values for both Chao1 and inverse Simpson are reported.

We did a classical multidimensional scaling followed by a multiple response permutation procedure (MRPP) using Bray–Curtis distance and 999 permutations to test for significant differences in microbial community composition above and below the oxycline. Methods such as non-metric multi-dimensional scaling were not applicable due to the large difference in number of samples and variables.

## Results

### Overall Microbial Community Composition

**FIGURE 1:**
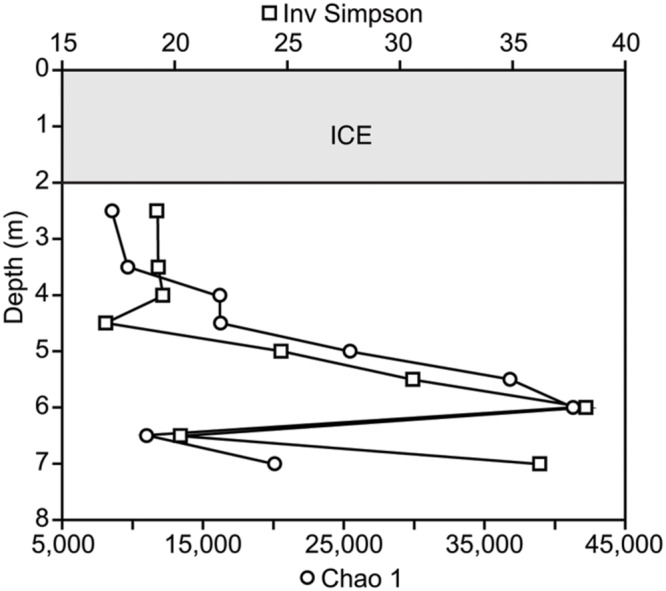
**Microbial richness and diversity throughout the water column using the Chao 1 estimator and inverse Simpson index based on the number of OTUs and their relative abundances detected in each sample**.

**FIGURE 2:**
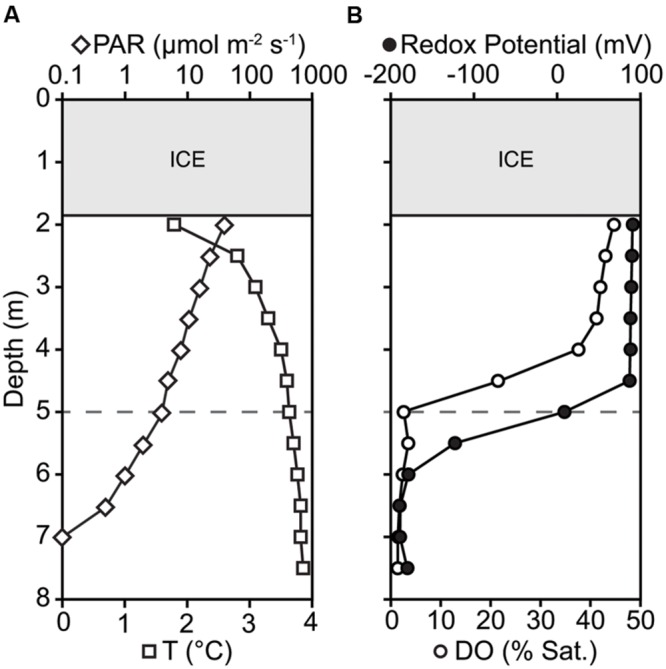
**Changes in **(A)** temperature (T), photosynthetically active radiation (PAR), **(B)** dissolved oxygen saturation (DO), and Redox Potential down the water column.** Oxycline is defined where DO saturation is <2%; marked by gray dashed horizontal line at 5.0 m depth.

**FIGURE 3:**
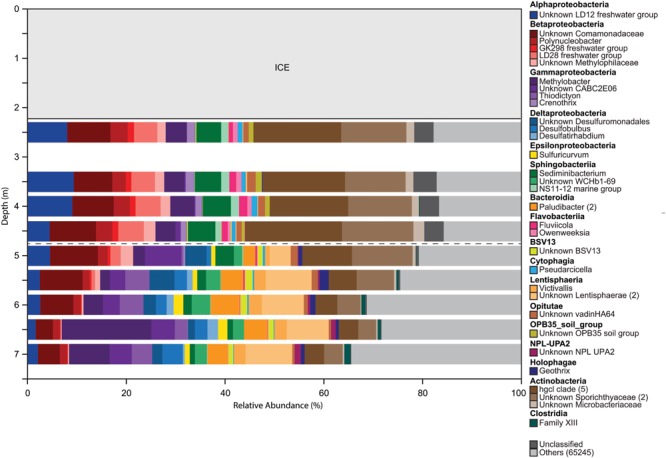
**Overall changes in microbial community composition based on changes in the relative abundances of 16S rRNA gene sequences classified into OTUs with 97% sequence similarity.** All OTUs with a relative abundance >1% are shown. OTUs with identical taxonomic identification (99–100%) were combined into the same taxonomic group and the number of OTUs included in each taxonomic group is indicated with the number given after the taxonomic name. If no number is given, the group only contains one OTU. OTUs with a relative abundance <1% are combined in the category ‘others.’ Dashed gray horizontal line shows the oxycline at 5.0 m.

The microbial community was diverse, with richness and diversity overall increasing with depth throughout the water column (**Figure 1**). We obtained overall 6,149,349 sequences after quality control and number of sequences obtained across samples ranged from 438,086 to 883,870. Community composition shifted significantly below 5.0 m depth (MRPP, *p* = 0.006). Richness estimates ranged from 8,557 OTUs at 2.5 m under the ice cover to over 41,308 OTUs at a depth of 6.0 m (Chao1 richness estimator, **Figure 1**). Under ice cover, suboxic conditions were observed decreasing from 45% at 2 m to 21.5% DO saturation at 4.5 m, and anoxic conditions (<2% DO) from 5.5 m down to the sediment/water interface (**Figure 2**). Herein, we will refer to 5.0 m as the oxycline. ORP directly correlated with DO, with a sharp decrease in ORP from 87 mV at 4.5 m to -123 mV at 5.5 m (**Figure 2**). Community composition of the abundant members appeared to respond strongly to redox conditions, shifting from a predominantly heterotrophic aerobic community in the suboxic waters to a predominantly heterotrophic anaerobic community in the anoxic waters (**Figure 3**). OTUs belonging to Sporichthyaceae, Comamonadaceae, and the LD12 freshwater group within the SAR11 Clade were abundant above the oxycline and higher abundances of OTUs belonging to the family Methylococcaceae, phylum Lentisphaerae, and PSB such as *Thiodictyon* were detected below the oxycline. Phototrophs such as cyanobacteria were present directly under the ice, where PAR was >10 μmol m^-2^ s^-1^, but had low relative abundance of below 0.001%. We detected Melainabacteria in particular at 6.5 and 7.0 m with abundances of 0.2%, a phylum related to Cyanobacteria but obtaining their energy by fermentation not photosynthesis. Above the oxycline more OTUs were present that had a relative abundance of over 5–6% and that below the oxycline fewer OTUs dominated the microbial community, but a larger number of rare OTUs resulted in higher overall richness.

### Methanogenesis and Methane Oxidation

**FIGURE 4:**
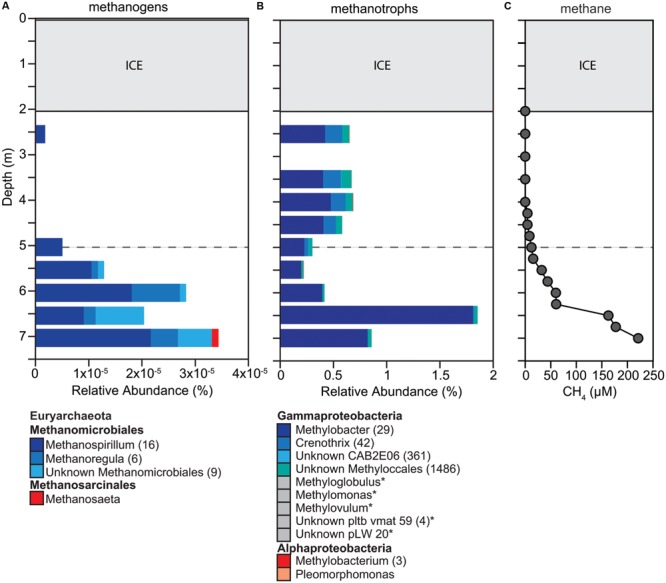
**Changes in composition of potential **(A)** methanogens and **(B)** methanotrophs down the water column, as well as **(C)** methane concentration throughout the water column. (A,B)** We determined taxonomic identification of the 16S rRNA genes classified into OTUs for each functional group using the SILVA database. OTUs with identical taxonomic identification (99–100%) were combined into the same taxonomic group and the number of OTUs included in each taxonomic group is indicated with the number given after the taxonomic name. If no number is given, the group only contains one OTU. Taxonomic groups with abundances too low to be visible on the graph are labeled with an ‘^∗^’ and marked with a gray box instead of a colored box. Dashed gray horizontal line shows the oxycline at 5.0 m.

The distribution of methanogens and methanotrophs was consistent with CH_4_ concentrations throughout the water column (**Figure 4**). The concentrations of CH_4_ ranged from 0.1 μM below ice cover to 225 μM at 7.0 m (**Figure 4C**). Above the oxycline, CH_4_ concentrations were <10 μM. Methanogens were detected throughout the water column but were most abundant below the oxycline with the highest relative abundance of 0.0034% at 7.0 m (**Figure 4A**). We detected a total of 48 OTUs all belonging to the Euryarchaeota. Most OTUs were classified within the order Methanomicrobiales (*Methanospirillum, Methanoregula*) and only a few of the OTUs belonged to the genus *Methanosaeta*. Overall, the relative abundance of potential methanogens was very low compared to other OTUs suggesting that methanogenesis under ice cover was restricted primarily to the sediments.

We detected a large diversity of potential methanotrophs throughout the water column (2,243 OTUs), representing one of the most abundant functional groups within the microbial community (>20%; **Figure 4B**). All of the methanotroph OTUs classified within the family Methylococcaceae (type I methanotrophs). The relative abundance of potential methanotrophs ranged from 5.34 to 13.08% with a significant increase at 6.5 m that can be attributed to the increase in relative abundance of one OTU within the genus *Methylobacter* (**Figure 4B**). Two OTUs made up 77% and four OTUs made up 86% of all OTUs detected, which suggests that at the time point sampled only a few OTUs accounted for most of the methanotrophs detected. Methanotrophs were detected throughout the water column but were in high relative abundance below the oxycline where DO saturation was below 2% suggesting that aerobic and anaerobic CH_4_ oxidation may be occurring in Potentilla lake.

### Sulfur Oxidation and Reduction

**FIGURE 5:**
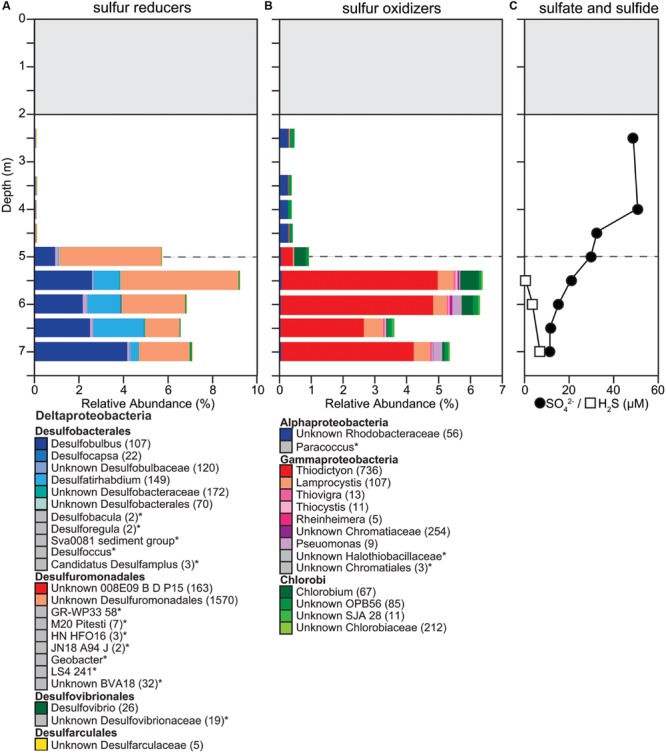
**Changes in composition of potential **(A)** sulfur reducers, **(B)** sulfur oxidizers, and **(C)** sulfate, sulfide concentrations down the water column. (A,B)** We determined taxonomic identification of the 16S rRNA genes classified into OTUs for each functional group using the SILVA database. OTUs with identical taxonomic identification (99–100%) were combined into the same taxonomic group and the number of OTUs included in each taxonomic group is indicated with the number given after the taxonomic name. If no number is given, the group only contains one OTU. Taxonomic groups with abundances too low to be visible on the graph are labeled with an ‘^∗^’ and marked with a gray box instead of a colored box. Dashed gray horizontal line shows the oxycline at 5.0 m.

Changes in relative abundance of sulfur oxidizers agreed with the concentrations of ΣH_2_S throughout the water column. The community composition of sulfur oxidizers can be divided into two zones, above and below the oxycline. Above the oxycline we observed an overall lower relative abundance of potential sulfur oxidizers (<1%), which is consistent with ΣH_2_S concentrations being below the detection limit (5 μM; **Figure 5**). The OTUs detected in the suboxic upper water column were identified as GSB within the order Chlorobiales and the PNSB family Rhodobacteraceae. Below the oxycline the relative abundance of PSB increased, with the community of sulfur oxidizers being dominated by *Thiodictyon* and *Lamprocystis* (**Figure 5**). We identified a total of 1,196 OTUs as PSB and all were classified as Chromatiaceae. The most abundant OTU was classified as *Thiodictyon* and had a relative abundance of 4% below the oxycline. This increase in the relative abundance of sulfur oxidizers below the oxycline and the shift in its community composition were consistent with chemical profiles. Increased ΣH_2_S concentrations below 5.5 m could support sulfur oxidation and a higher relative abundance of sulfur oxidizers. In addition, the increase in ΣH_2_S corresponded with a decrease in DO, as well as a decrease in PAR. The combination of chemical and physical changes below 5.5 m was coincident with a shift in community abundance to sulfur oxidizers dominated by PSB.

Potential sulfur reducers were only present at very low abundances in the suboxic water column (0.05–0.07%) and also had a significant increase in relative abundance below the oxycline (5.5–8.7%; **Figure 5**). With 1,999 OTUs, we detected a large diversity of potential sulfur reducers. The more abundant OTUs of potential sulfate reducers fall within the genus of Gram-negative metal and sulfur-reducing *Geobacter*, the sulfate-reducing *Desulfobulbus*, and sulfate-, sulfite-, and sulfur-reducing Desulfobacteraceae. Sulfate was present throughout the water column, decreasing down the water column from 49 μM below the ice to 11 μM above the sediment. The decrease in SO_4_^2-^ concentrations corresponded with both DO and ORP reductions with depth (**Figures 2** and **5**). Changes in relative abundance of potential sulfate reducers corresponded with a decrease in SO_4_^2-^ concentrations observed below the oxycline. Below the oxycline, SO_4_^2-^ and ΣH_2_S concentrations displayed opposite trends suggesting that sulfate reduction was producing H_2_S.

### Denitrification, Ammonia Oxidation, and Nitrogen Fixation

**FIGURE 6:**
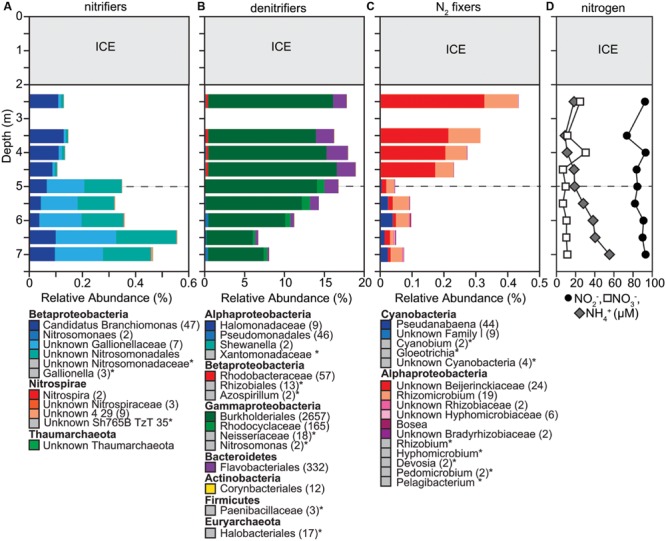
**Changes in composition of potential **(A)** ammonia oxidizers, **(B)** denitrifiers, and **(C)** nitrogen fixers, as well as **(D)** concentrations of nitrite, nitrate, and ammonia down the water column. (A–C)** We determined taxonomic identification of the 16S rRNA genes classified into OTUs for each functional group using the SILVA database. We summed up the relative abundances of all OTUs classified within the same order or classes. If a genus name is shown, it means that that was the only genus present in a particular order or class. The number of OTUs included in each taxonomic group is indicated with the number given in parentheses after the taxonomic name. If no number is given, the group only contains one OTU. Taxonomic groups with abundances too low to be visible on the graph are labeled with an ‘^∗^’ and marked with a gray box instead of a colored box. Dashed gray horizontal line shows the oxycline at 5.0 m.

Potential denitrifiers composed a large part of the overall microbial community (7–16%; **Figure 6**). The distribution of potential denitrifiers followed the concentration of NO_2_^-^ and NO_3_^-^ through the water column. Concentrations of NO_2_^-^ (median 90 μM) were approximately eight times greater than NO_3_^-^ (median 10.5 μM). Relatively uniform concentrations of NO_2_^-^ and NO_3_^-^ were observed down the water column except for increases at 2.5 m and 4.0 m. These sudden increases in both NO_2_^-^ (20%) and NO_3_^-^ (>50%) corresponded with a slight increase in the relative abundance of two potential denitrifiers. One OTU within the family Comamonadaceae in the order Burkholderiales decreased by ∼12% between 2.5 and 3.5 m and then increased by ∼12% to 4.0 m. Similarly, *Polynucleobacter* within the order Burkholderiales decreased by ∼37% between 2.5 and 3.0 m and increased by ∼15% between 3.5 and 4.0 m and then decreased by a 40% below 5.0 m. Except for a few OTUs changing in relative abundance (Comamonadaceae within the order Burkholderiales, Rhodocyclaceae within Betaproteobacteria), the relative abundance of most OTUs identified as potential denitrifiers stayed consistent throughout the water column (**Figure 6**). This result differs from the other functional groups described above where we observed a clear shift in community composition below the oxycline.

Potential ammonia oxidizers made up a low percentage (0.1–0.34%, **Figure 6**) of the overall microbial community composition. Of the more abundant potential ammonia oxidizers, all except one classified within the order Nitrosomonadales. One OTU classified within Nitrospirales, an order that contains several genera, the largest being *Nitrospira*, which is known for converting NO_2_^-^ to NO_3_^-^ (Maixner et al., 2006). Concentrations of NH_4_^+^ increased with depth, ranging between 11 and 55 μM. The relative abundance of potential ammonia oxidizers followed this increase in NH_4_^+^ with depth suggesting that the increase in NH_4_^+^ may result in a higher activity of ammonia oxidation, but can be attributed to the increase in two OTUs classified within the Gallionellaceae. Thus, similar to potential denitrifiers, the composition of ammonia oxidizers stayed stable throughout the water column and changes in community composition can be attributed to changes in the relative abundances of only a few OTUs.

Similar to potential ammonia oxidizers, the abundance of potential nitrogen fixers was low and ranged between 0.15 and 0.69%, which fits the general pattern that phototrophic bacteria were only present in low abundance even directly under the ice where PAR > 10 μmol m^-2^ s^-1^. The community composition of potential nitrogen fixers differed markedly above (Rhizobiales dominates) and below (*Chlorobium* dominates) the oxycline and one OTU classified as Cyanobacteria decreased in its relative abundance 100-fold between 2.5 (0.02%) and 6.5 m (0.0002%; **Figure 6**). Interestingly we detected 135 OTUs classified as Melainabacteria. Their relative abundance increased with depth to about 0.44%. This sibling phylum to cyanobacteria has been recently characterized as non-photosynthetic, anaerobic, and obligate fermentative (Di Rienzi et al., 2013).

## Discussion

### Sulfur Oxidation and Reduction

We detected significant abundances of PSB around the oxycline. All OTUs identified as PSB were classified within the Chromatiaceae, which are known to be able to deposit elemental sulfur within their cells (Imhoff, 2006). Overall PSB were the most abundant potential sulfur oxidizer compared to both GSB and PNSB, particularly below the oxycline. The abundance and diversity of PSB had similarities (Tonolla et al., 1999; Rogozin et al., 2009, 2012; Peduzzi et al., 2011) and differences (Karr et al., 2003; Koizumi et al., 2003; Taipale et al., 2011; Comeau et al., 2012) with other studies under ice cover. Rogozin et al. (2009, 2012) found high densities of PSB, predominantly *Lamprocystis*, in two meromictic, seasonally ice-covered lakes in Siberia and low abundance of GSB or no GSB. In one of the lakes, the density of PSB was sufficiently high in some years to induce self-shading. In perennially ice-covered Lake Fryxell in Antarctica, Karr et al. (2003) detected a large diversity of phototrophic purple bacteria, but the authors only detected sequences similar to those of known PNSB. In a perennially ice-covered, meromictic lake in the Canadian Arctic, 40% of sequences belonged to the phylum Chlorobi, a group of GSB and a few to PNSB just below the chemocline, but PSB were not detected (Comeau et al., 2012). In contrast, we detected all three groups of sulfur oxidizing bacteria and found both coexistence and zonation throughout the water column. PNSB such as Rhodobacteraceae were more abundant above the oxycline and PSB and GSB coexisted below the oxycline.

Adaptive divergence of pigments, with differentially tuned absorbance spectra, has been suggested as an important factor in the diversity of phototrophic microorganisms in aquatic environments (Stomp et al., 2004, 2007). Development of mutualistic vertical structures in microbial communities of perennially frozen meromictic lakes in Antarctica may be partly driven by availability of light, key nutrients, and phage-mediated predation (Lauro et al., 2011). These are likely factors in the vertical structure of plankton in seasonally ice-covered lakes of the Arctic as well. For example, PSB and GSB differ in primary pigments and in the wavelengths of peak absorbance. PSB peak absorbances are between 440–486 nm and 780–860 nm, while peaks for GSB are 400–460 nm and 680–720 nm (Stomp et al., 2007). Light attenuation through snow, ice, and water is greater for longer wavelengths. Thus, we would expect the relative abundance of PSB to be greater at somewhat shallower depths. However, both PSB and GSB carry out anoxygenic photosynthesis using reduced sulfur and are likely inhibited by elevated DO present above the oxycline, and are adapted to low light conditions (Vila and Abella, 2001). GSB have been reported to grow under near-infrared light at photon fluxes <10 μmol m^-2^ s^-1^ (Saikin et al., 2014).

Major shifts in dominant species from season to season are expected in highly oligotrophic ice-covered systems (Lauro et al., 2011; Wilkins et al., 2013). In 2013, Potentilla lake was ice-covered, but had no snow cover. Our microbial community analysis showed that 30% of the overall microbial community was attributed to a single OTU of the PSB *Lamprocystis* at a depth of 5 m. In 2014, Potentilla lake had a significant snow cover in addition to the ice cover and PSB only contributed about 8% of the overall microbial community at 5 m. The additional snow cover changed light penetration and that likely resulted in a reduction in PSB. However, lower light levels may also have driven changes in the relative abundances of sulfur oxidizers. Further, PSB can oxidize various reduced sulfur species including sulfide, thiosulfate (Holkenbrink et al., 2011), as well as oxidize elemental sulfur (Dahl, 2008; Frigaard and Dahl, 2009). Similarly, GSB typically oxidize sulfide and thiosulfate to sulfate but lost essential genes necessary for elemental sulfur oxidation (Gregersen et al., 2011). Hydrogen sulfide concentrations dropped below the oxycline and if elemental sulfur was indeed present below the oxycline it could have resulted in PSB outcompeting GSB. However, more likely reasons seem to be differences in methodology used to determine the presence of different photosynthetic sulfur oxidizers and differences in the physical structure of the lakes sampled. Most studies use differences in BChl *a* versus BChl *d* to determine the amount of PSB and GSB present, respectively. In comparison, we used a next generation sequencing technique, which may have a higher resolution in detecting rare sulfur oxidizers. Further, dimictic lakes such as the one sampled in this study mix twice a year and while Potentilla lake has ice cover for about 10 months a year it is not ice-covered in the summer. This suggests that seasonally dimictic Arctic lakes may be less stable systems compared to meromictic perennially frozen lakes. Thus, we postulate that PSB are more competitive than GSB in these less stable lake systems while GSB are more competitive in stable lake systems such as perennially frozen meromictic lakes in Antarctica (Lauro et al., 2011) and that PSB potentially are an important component in sulfur oxidation in Arctic dimictic lakes.

Below the oxycline we observed an inverse relationship in H_2_S and SO_4_^2-^, with H_2_S increasing down the water column corresponding with the continuous decrease in SO_4_^2-^ concentration, consistent with a significant increase in the presence of potential sulfur reducers. Overall, the groups of potential sulfate reducers identified were similar to those previously identified in perennially frozen, Antarctic Lake Fryxell (Karr et al., 2005). Previous studies have shown strong competition between sulfate reducers and methanogens in Arctic lake sediments (Lovely et al., 1982; Purdy et al., 2003) and found that when SO_4_^2-^ is not limiting, sulfate reducers inhibit methanogenesis by decreasing the hydrogen partial pressure below levels methanogens can utilize (Lovely et al., 1982). In Potentilla lake, potential sulfur and sulfate reducers were significantly more abundant than methanogens in the lower water column. Even at low SO_4_^2-^ concentrations (<50 μM), a large relative abundance of potential sulfur and sulfate reducers (5–9%) were supported that may outcompete methanogens. In other Arctic lakes, increasing temperatures and reduced ice cover have resulted in an increase of SO_4_^2-^ and sulfur accumulation in the sediments (Drevnick et al., 2010). Increasing SO_4_^2-^ under a warming climate may result in greater sulfur reduction rates, suppressing methanogenesis, and thereby decreasing the concentration of CH_4_ in Arctic lakes.

### Methane Oxidation and Methanogenesis

Potential methanotrophs composed a large proportion of the microbial community. Below 6.5 m concentrations of CH_4_ were comparatively high (160–220 μM) and DO saturation was <2%, suggesting that methane oxidation was occurring either aerobically or anaerobically. Several studies have shown anaerobic methane oxidation (AOM) coupled to sulfate (Boetius et al., 2000; Deutzmann and Schink, 2011; Milucka et al., 2012), iron, and manganese reduction (Beal et al., 2009) in marine systems, and coupled to nitrate and nitrite reduction in a bioreactor system (Haroon et al., 2013) and an enrichment culture from a peatland (Zhu et al., 2012). The known anaerobic methanotrophic Archaea (ANME) are related to the genus of methanogens *Methanococcoides* (Knittel et al., 2005; Schleper et al., 2005; Cui et al., 2015) and distantly to Methanosarcinales and Methanomicrobiales. We detected two OTUs of very low abundance within the Methanosarcinales, but several OTUs within the order of Methanomicrobiales. The ANMEs distantly related to the Methanomicrobiales were shown to carry out sulfate dependent anaerobic methane oxidation (Knittel et al., 2005). However, compared to the relative abundance of the aerobic methanotrophs we detected, the relative abundance of ANMEs was low. This suggests AOM may be occurring, but at low rates. More likely, AOM was occurring in the sediments and DO levels at that depth were high enough for methane oxidation to be carried out by microaerophilic methanotrophs (Ren et al., 1997; van Bodegom et al., 2001).

The two most abundant aerobic methanotrophic OTUs detected were classified as *Methylobacter* and within the same family Methylococcaceae. Both of these aerobic methanotrophs were highly abundant below the oxycline (>20%) and made up the majority of the methanotrophic community in that part of the water column. The high relative abundances of aerobic methanotrophs and that DO was <2% below the oxycline suggest that these methanotrophs are microaerophilic (Ren et al., 1997). The OTU classified within *Methylobacter* was also abundant in the upper water column above the oxycline suggesting that CH_4_ was being oxidized efficiently throughout the water column under ice cover. In other boreal and polar lakes, the flux of CH_4_ during ice-melt associated with spring overturn has been shown to be a significant input of CH_4_ into the atmosphere in the spring (Phelps et al., 1998; Juutinen et al., 2009; López Bellido et al., 2009; Song et al., 2012). The decrease in CH_4_ concentration up the water column in Potentilla lake may be attributed to consumption by both AOM and microaerophilic methanotrophs, thereby mitigating the potential emission of CH_4_ to the atmosphere during ice-melt and spring overturn.

Potential methanogens were most abundant below the oxycline but their overall relative abundance was very low throughout the water column. Quality and quantity of nutrients could potentially be low at the end of the ice-covered season because ice cover decreases input of nutrients from the terrestrial surroundings and atmosphere (Bertilsson et al., 2013). More likely, methanogenesis occurs in lake sediments where more favorable electron acceptors are absent (Bertilsson et al., 2013). This would suggest that the source of CH_4_ observed in the water column originated in the sediments, and that the CH_4_ in the lower water column was oxidized once oxygen levels were sufficient for methane oxidation or by AOM. The types of methanogens identified, such as Methanosaeta and Methanomicrobiales, are similar to methanogens detected in sediments of a seasonally ice-covered, dimictic, oligotrophic lake in Germany (Conrad et al., 2007) but differ from the ones in perennially frozen, meromictic, Antarctic Lake Fryxell (Karr et al., 2006). Lake Fryxell is a highly sulfidic and stratified lake compared to Potentilla lake, suggesting that methanogenic communities in Potentilla lake are determined by a set of factors including duration and amount of snow and ice cover, amount of nutrients available in addition to polar climate.

### Ammonia Oxidation and Denitrification

The relative abundance of potential ammonia oxidizers was stable throughout the water column, but potential nitrite oxidizers increased in abundance below the oxycline. Ammonium is an important nitrogen source for other microorganisms such as methane and sulfur oxidizers, and the low relative abundance of potential ammonia oxidizers compared to methanotrophs and PSB suggests that ammonia oxidizers may not be competitive for ammonium. Pouliot et al. (2009) found strong vertical differentiation of archaeal ammonia oxidizers in an Arctic marine-influenced meromictic lake, with the largest abundances around the oxycline. The authors suggest that Archaea may be important contributors to ammonia oxidation, which is consistent with studies in other systems that showed the importance of Thaumarchaeota as ammonia oxidizers (Church et al., 2003; Ochsenreiter et al., 2003; Zhang et al., 2008, 2010; Lehtovirta et al., 2009; Dodsworth et al., 2011; Stahl and de la Torre, 2012). However, in our study OTUs classified within the Thaumarchaeota were very rare (∼0.0001%). Nitrite oxidation should be supported throughout the water column as nitrite concentrations were higher compared to other ice-covered lakes (Auguet and Casamayor, 2013). *Gallionella*, was the most abundant potential nitrite oxidizer below the oxycline consistent with Alawi et al. (2007) who cultivated a nitrite oxidizer most closely related to the genus *Gallionella*. Potential nitrite oxidizers were particularly low in abundance above the oxycline suggesting that aerobic nitrite oxidizers could not compete for oxygen with aerobic ammonia oxidizers above the oxycline. However, below the oxycline, potential nitrite oxidizers increased, suggesting they were competitive for NO_2_^-^ (Nakano and Zuber, 2004). The combination of high NO_2_^-^ levels, increased NH_4_^+^ concentrations and the presence of OTUs classified as *Anammoximicrobium* (0.01–0.03%, data not shown) below the oxycline suggest the potential for anaerobic ammonia oxidation. Anaerobic ammonia oxidation converts NO_2_^-^ and NH_4_^+^ into nitrogen gas and is responsible for up to 50% of nitrogen losses in marine environments (Kuenen, 2008). Concentrations of NH_4_^+^ and relative abundances of potential ammonia and nitrite oxidizers may be currently low in Potentilla lake because of limited surface runoff. However, the amount and type of surface runoff may change as climate change alters precipitation, snowmelt, and permafrost thaw (Anderson et al., 2002; Vincent et al., 2012).

The potential denitrifiers we detected are known for complete and incomplete denitrification. For example, representatives of the group Comamonadaceae have been shown to carry out complete denitrification in activated sludge systems (Etchebehere et al., 2001; Ginige et al., 2005). This suggests that while Comamonadaceae, one of the most abundant OTUs of potential denitrifiers we found, may be using aerobic respiration in the upper water column above the oxycline, they could also be important for denitrification below the oxycline. Incomplete denitrification is known to produce nitrous oxide, a potent greenhouse gas (Fernandes et al., 2010; Abed et al., 2013). However, we detected OTUs that were classified within Alcaligenaceae, a group of denitrifiers that have been shown to contain the *nos*Z gene that encodes for the nitrous oxide reductase (Velusamy and Krishnani, 2013). This enzyme reduces nitrous oxide to dinitrogen (Henry et al., 2006), thus potentially resulting in complete denitrification of nitrate and reducing the buildup of nitrous oxide in Potentilla lake. The presence of NO_3_^-^ inhibits NO_2_^-^ reduction (Neubauer and Götz, 1996) resulting in a buildup of NO_2_^-^, which may account for NO_2_^-^ levels that exceeded NO_3_^-^ levels by a factor of eight. The decrease in NO_3_^-^ levels below 4.0 m is reflected in the decrease in the relative abundance of potential denitrifiers. Voytek et al. (1998) found that NO_3_^-^ levels were always below detection limit in a permanently frozen lake in Antarctica. Compared to this study, NO_3_^-^ levels in Potentilla lake were high, which may be an explanation for the high relative abundances of potential denitrifiers. The large relative abundances of potential denitrifiers indicate that denitrifiers may be carrying out an important part of nitrogen cycling. Understanding denitrification dynamics in Arctic lakes may be particularly important as terrestrial run-off due to warming temperatures is predicted to rise in Arctic landscapes (Vincent et al., 2012).

### Microbial Response to Environmental Change

We also sampled Potentilla lake under ice cover in 2013 and found similar geochemical conditions but differences in the microbial populations. In late winter 2013, PSB composed a large proportion of the overall microbial community with one OTU identified as *Lamprocystis*, composing >30% of the total abundance at 6.0 m depth. In the following winter 2014, the overall abundance of PSB was lower and, while *Lamprocystis* was present, the most abundant PSB OTU was classified as *Thiodictyon*, which suggests that microbial community members may cycle between abundant and rare. The dynamic year-to-year changes in abundance are consistent with field and modeling studies of Ace Lake in Antarctica (Lauro et al., 2011).

It should be noted that for most functional groups detected in 2014, most OTUs were rare (<1%). For example, four out of 1778 OTUs identified as potential methanotrophs had a relative abundance >1%. This does not indicate that the rare OTUs were not active. Dormant and rare members in the communities can be disproportionally active (Jones and Lennon, 2010). Additionally, a large proportion of the active bacterial community can cycle between abundant and rare (Campbell et al., 2011), and rare members can become active spontaneously (Epstein, 2009) or due to environmental changes. The differences in microbial community composition observed between 2013 and 2014 may be occurring regularly in response to environmental factors such as the extent of snow pack development. In 2013, the lake had no snow cover, whereas in 2014, it was covered by ∼20 cm of snow. The associated differences in light penetration may account for the changes in PSB community composition between 2013 and 2014, illustrating the importance of light as a microbial driver for phototrophic growth in oligotrophic polar lakes (Stomp et al., 2007; Wilkins et al., 2013). Under accelerated warming in the Arctic, ice-cover thickness and duration are likely to decrease and snow-cover regimes will change. We suggest that these changes will likely drive significant variations in microbial ecologies related to differences in light penetration and thermal structure of Arctic lakes.

## Conclusion

We showed that microbial communities changed consistently with geochemical properties throughout the water column under ice in Potentilla lake. The microbial community was diverse and changed markedly in composition above and below the oxycline with a surprisingly low relative abundance of phototrophs. For the first time, we showed the importance of PSB as potential sulfur oxidizers in Arctic dilute dimictic lakes under seasonal ice cover. Concentrations of CH_4_ decreased up the water column, which we attribute to consumption by both AOM and microaerophilic methanotrophs, thereby mitigating the potential emission of CH_4_ to the atmosphere during spring overturn and ice-melt. The high concentrations of NO_3_^-^ and NO_2_^-^ indicate that denitrifiers may be carrying out an important part of nitrogen cycling. Together with previous studies, we show that the microbial community dynamics under ice-cover are complex. Broad-scale surveys, involving metatranscriptomics and metaproteomics in combination with detailed geochemical analysis, are needed to identify local and regional processes and their interactions that control the microbial dynamics of these types of lakes and their response to climate change (Reuss et al., 2013).

## Author Contributions

US was involved in the experimental design of this study, advised and trained the undergraduates to determine microbial community composition, was heavily involved in the data analysis, and was the main writer of this manuscript. SC designed the experiment, carried out the field work, did all the geochemical analysis, and was heavily involved in data analysis and writing of the manuscript. CH did all the bioinformatic analysis of the MiSeq sequences, was involved in data analysis and writing of the manuscript. LP was involved in the experimental design, field work, data analysis, and writing of the manuscript. JW was involved in the experimental design, field work, geochemical analysis, data analysis, and writing of the manuscript.

## Conflict of Interest Statement

The authors declare that the research was conducted in the absence of any commercial or financial relationships that could be construed as a potential conflict of interest.

## Footnotes

1 http://www.earthmicrobiome.org

2 http://www.earthmicrobiome.org/emp-standard-protocols/16s/
